# Book Review: Systems and Synthetic Biology

**DOI:** 10.3389/fbioe.2017.00050

**Published:** 2017-08-18

**Authors:** Vijai Singh

**Affiliations:** ^1^Synthetic Biology Laboratory, Department of Microbiology, School of Biological Sciences and Biotechnology, Institute of Advanced Research, Koba Institutional Area, Gandhinagar, India

**Keywords:** systems biology, synthetic biology, non-coding RNA, synthetic network, drugs

This book has been written to offer a complete and better understanding as well as a deeper exploration of systems and synthetic biology research on a worldwide stage. It covers the recent advances and developments in both areas, offering insights into the core disciplines of systems biology and synthetic biology, as well as into their overlap (Singh and Dhar, 2015). During the mid-1990s, systems biology was developed with the aim to study various systems of biological components, which included those of molecules, cells, and organisms. Living systems are dynamic and complex, and consequently their overall behavior is often hard to predict from the properties of their individual parts, if possible. Quantitative measurement techniques have been used for the study of the behavior of groups of interacting components. These have involved a wide range of technologies that have spanned the fields of genomics, bioinformatics, proteomics, and mathematical and computational modeling. Following this progression, scientists have since become able to test dynamic behaviors with better insight. Thereafter, scientists have designed and constructed genetic networks that have permitted behavioral predictions for living cells, and this has emerged as synthetic biology. Synthetic biology possesses the potential to solve, and in some cases has solved, questions in important issues that include food supply, drug synthesis, chemical development and production, and environment and energy concerns (Khalil and Collins, 2010; Singh, 2014).

This book is a timely compilation of highly focused reviews written by eminent scientists in the fields of systems and synthetic biology. It is aimed for advanced students and researchers with a firm grounding and understanding in the basic concepts. This book has been divided into two parts (I and II). The Part I consists of 11 chapters on systems biology, and the second part contains 10 chapters on synthetic biology. Each chapter covers the background, research challenges, recent developments, and future outlooks. For the first part, Chapter 1 gives the basic introduction to systems biology, and describes the origin and the acceptance of systems biology as a field, and details the opportunities and challenges in the generation of models and their applications. Chapters 2–3 describe the recent advances in modeling and simulation approaches for studying the complex natural phenomenon and the dynamics of biological processes. Chapter 4 describes how continual increases in the quantity and quality of genomic data can allow us to identify eukaryotic promoters *in silico*. When compared to experimental screening, this offers an easy, fast, and reliable alternative to wet laboratory work. Chapters 5–7 describe the different modeling approaches for transcriptional regulation and the analysis of complex networks for the better understanding of biological systems. Chapter 8 covers the systems biology approaches for studying infectious diseases. This can be valuable for identifying potential drug targets, generating hypotheses, and predicting the causes and outcomes of disease.

Chapter 9 describes the network analysis of interactions that are involved in disease pathophysiology and drug response. This can allow the integration of the systems-level understanding of drug action with genetic information, to facilitate the development of truly personalized medicine. Chapter 10 describes the switching mechanism in the p53 regulatory network, whereby p53 is one of the most important signaling molecules that regulate a wide range of metabolic biochemical pathways. It plays a key role in cellular homeostasis and protects cellular integrity. It also controls cellular transition from a normal to a cancerous state. Chapter 11 covers the systems biology of microRNAs, which are small non-coding RNAs that play key roles in regulation in all forms of life. It has been experimentally demonstrated that microRNAs can control systems, and therefore they can determine both genotype and phenotype.

In Part II, 10 chapters are dedicated to synthetic biology. Chapter 12 briefly describes and lays out the foundations of synthetic biology. Chapter 13 describes how promoter structure will affect and regulate gene expression. These have been designed in order to regulate the quantity of genetic expression in *Escherichia coli* and *Saccharomyces cerevisiae*. Chapter 14 describes synchronous sequential computations with biomolecular reactions. Of immense interest, this topic and its findings have the potential to develop synthetic circuits for biochemical sensing and drug delivery. Chapter 15 describes the design of zinc finger proteins for their applications in synthetic biology toward genome editing, as well as biomedical and therapeutic use. Chapter 16 describes the use of synthetic biology for the development of drugs and also of designer crops, for better performance and higher production of desired molecules. It also discusses a number of important ethical and regulatory issues. Chapter 17 covers the recent advances in the synthetic biology toolkit for the acceleration of the design, construction, and characterization of synthetic parts, devices, and systems for biotechnological applications. Chapter 18 describes the recent advances in metabolic engineering for the production of antibiotics toward their increased production, and novel targeting methods for the development of new drugs. Chapter 19 describes the engineering art of DNA origami, detailing its origin and functions toward the folding and shaping of DNA. Chapter 20 describes the building of peptides from non-coding DNA (Dhar et al., 2009) that have the potential to offer high levels of functional activity for the targeting of neurological diseases and cancers, as well as other diseases. The final chapter, Chapter 21, introduces recent innovative developments in the field of synthetic biology.

These fields remain fast growing and greatly expanding research areas, which have led to a wealth of information and recent published literature that update the overall knowledge. This book covers most of the recent and technical developments in systems and synthetic biology. By compiling and summarizing our current knowledge on systems and synthetic biology, the authors have provided us with a rich literary material of excellent depth, coverage, and clarity. Altogether, this book is an excellent basis from which scientific knowledge grow and widen in the fields of systems and synthetic biology. It would offer great benefit in the hands of anybody with a sound interest in these matters, from researchers and students to current professionals looking to capitalize on this ever-growing and continually adventurous cutting edge of the biological sciences. This book is ideal for highlighting the progress in how these scientific disciplines are addressing our real world issues in the subjects of healthcare, energy, and the environment.

## Author Contributions

VS designed and wrote the manuscript.

## Conflict of Interest Statement

The author declares that the research was conducted in the absence of any commercial or financial relationships that could be construed as a potential conflict of interest.
